# Advancing Lie Detection by Inducing Cognitive Load on Liars: A Review of Relevant Theories and Techniques Guided by Lessons from Polygraph-Based Approaches

**DOI:** 10.3389/fpsyg.2013.00014

**Published:** 2013-02-01

**Authors:** Jeffrey J. Walczyk, Frank P. Igou, Alexa P. Dixon, Talar Tcholakian

**Affiliations:** ^1^Psychology and Behavioral Sciences, Louisiana Tech UniversityRuston, LA, USA

**Keywords:** cognition of deception, cognitive lie detection, rehearsed deception, polygraph, inducing cognitive load

## Abstract

This article critically reviews techniques and theories relevant to the emerging field of “lie detection by inducing cognitive load selectively on liars.” To help these techniques benefit from past mistakes, we start with a summary of the polygraph-based Controlled Question Technique (CQT) and the major criticisms of it made by the National Research Council (2003), including that it not based on a validated theory and administration procedures have not been standardized. Lessons from the more successful Guilty Knowledge Test are also considered. The critical review that follows starts with the presentation of models and theories offering insights for cognitive lie detection that can undergird theoretically load-inducing approaches. This is followed by evaluation of specific research-based, load-inducing proposals, especially for their susceptibility to rehearsal and other countermeasures. To help organize these proposals and suggest new direction for innovation and refinement, a theoretical taxonomy is presented based on the type of cognitive load induced in examinees (intrinsic or extraneous) and how open-ended the responses to test items are. Finally, four recommendations are proffered that can help researchers and practitioners to avert the corresponding mistakes with the CQT and yield new, valid cognitive lie detection technologies.

The seemingly disparate fields of “polygraph-based lie detection” and “research and theory on social-cognitive aspects of deception” seldom communicate. Still, lessons from the former may benefit attempts made from the latter perspective to detect lies. A goal of this critical review is to advance a new research area of the social-cognitive perspective, “lie detection by inducing cognitive load selectively on liars,” to develop on valid theoretical grounds and avoid other pitfalls that hampered the Controlled Question Technique (CQT), a questioning paradigm used with the polygraph. To this end, the CQT is summarized and major criticisms of it made by the National Research Council (2003) are shared. Some of them are that it is not based on a valid theory and is highly susceptible to countermeasures. Also summarized is the more successful polygraph-based Guilty Knowledge Test (GKT, a.k.a. the Concealed Knowledge Test), which overcomes many of these concerns (Lykken, 1998). With these lessons in mind and to help load-inducing lie detection efforts to develop on valid theoretical grounds, the critical review begins with discussion of models and theories relevant to the cognition of deception for their insights on cues to deception. Next, we consider the specific proposals appearing in the literature that try to make it cognitively more difficult to lie than to tell the truth, especially for their susceptibility to countermeasures. Then, a taxonomy of load-inducing lie detection is presented to organize these proposals and open up new research avenues. Coming full circle, we conclude with four recommendations for researchers and practitioners to avoid the corresponding problems with the CQT.

## Summary of Polygraph-Based Lie Detection: Its Uses, Pitfalls, and Successes

This section is not part of the review. Rather, it is a summary of certain aspects of polygraph-based lie detection. Critical reviews in this area are available elsewhere (e.g., Lykken, 1998; National Research Council, 2003). The *polygraph* is a device that continuously records psycho-physiological arousal as assessed by pulse rate, blood pressure, respiration rate, and skin conductivity, which has been applied to uncover deception. The most common questioning paradigm used with it for detecting lies is the CQT. In a typical test, an examinee is given a pretest interview for gathering information that can serve as the basis for control questions. Once questions are chosen, the examiner will preview them with the examinee to ensure that the questions are understood and do not surprise the examinee when asked later. During the exam, irrelevant questions are asked such as “What is your name?” along with control questions that most people tend to lie to. For example, “Have you ever stolen anything from the workplace?” Finally, relevant questions probe the issue central to the exam (e.g., “Did you kill …?”). The questions usually elicit brief answers. A liar is hypothesized to show more arousal to relevant questions than to control questions, whereas an innocent individual (truth teller) should show more arousal to control questions than to relevant questions (Lykken, 1998). Law enforcement and federal agencies in the USA use the CQT as a screening device for hiring and retaining employes and as a tool for criminal investigations. The CQT has been used to verify victim’s statements, evaluate the veracity of witnesses, and to exonerate suspects. Still, test results are largely inadmissible in US courtrooms (National Research Council, 2003).

The validity of the CQT has been challenged in a 2003 report by a distinguished panel of scientists of the National Research Council, which reviewed all available scientific studies and offered several criticisms. Among the most serious, the administration procedures for the CQT have not been standardized. The paradigm has a high rate of false positives (honest individuals misclassified as liars), is highly susceptible to countermeasures, and the results of examinations are subjectively scored. The criticism most germane here is that *the CQT is not based on a theory of deception that has been validated*. For instance, an assumption central to the CQT, that lying causes more sympathetic nervous system arousal than truth telling, is unsubstantiated. The panel called for research on alternatives. Many of these criticisms are translated later in this article into specific recommendations for advancing cognitive load-inducing lie detection techniques in ways that overcome these criticisms.

Partly in response to the validity concerns with the CQT, the GKT was proposed. It is a questioning paradigm that can be used with the polygraph to uncover the false denials of examinees by exposing whether they possess “guilty knowledge”, presumably resulting from their participation in a crime (Lykken, 1998). During a GKT, the examinee is presented with multiple-choice questions, each having one relevant alternative (correct answer) and several neutral alternatives (plausible distractors). The latter should be chosen such that an innocent person could not discriminate them from the relevant alternative (Lykken, 1998). An example of a relevant question is “How was the victim killed?” with the response alternatives of “shot,” “stabbed,” “struck,” “strangled,” or “poisoned.” This question could be re-asked multiple times, along with other questions probing different aspects of a crime scene. The examinee does not even need to answer. If heightened arousal occurs consistently to relevant responses, then the examinee may be concealing knowledge as the perpetrator. The GKT assumes that innocent examinees could not have acquired guilty knowledge indirectly and that guilty examinees encoded guilty knowledge and have retained it (Elaad, 1990).

Some validity concerns with the CQT were resolved in the GKT, including more standardization of the procedure, more appropriate control alternatives, fewer false positives, and a stronger theoretical basis (Lykken, 1998; Carmel et al., 2003). Also, beyond the psycho-physiological measures of the polygraph, guilty knowledge has been demonstrated with the diverse cues of response time (Seymour et al., 2000; Seymour and Kerlin, 2008; Seymour and Fraynt, 2009), event-related potentials (Rosenfeld et al., 1988, 2006), and pupil dilation (Dionisio et al., 2001), among others. The relative success of the GKT also offers lessons for the development of load-inducing lie detection techniques, especially that they should be based on a valid theory. Still, the GKT is limited in the deception it can uncover to the false denials of those possessing guilty knowledge.

## Models/Theories Advancing Understanding of the Cognition of Deception

Recalling that the CQT is not based on a validated theory of deception, we next review models and theories offering insights on the cognition of deception to help new load-inducing lie detection techniques to advance on solid theoretical ground. As will be discussed later, most of them lack such a foundation. Some accounts were proposed to explain social aspects of deceit, but offer important cognitive insights.

### Selected accounts undergirding the guilty knowledge test

Various theoretical accounts of how the GKT works have been proposed. Two are particularly relevant to the cognition of deception. The first, Orienting Response Theory, focuses on attentional processes. According to it, individuals tend to orient and attend carefully to environmental stimuli that are novel or emotionally significant to them, thereby preparing themselves to respond adaptively as necessary (Sokolov, 1963). Applied to the GKT, an orienting response naturally occurs in guilty examinees on exposure to relevant knowledge, as evidenced, for instance, by a lowering of heart rate, but not to neutral alternatives (Verschuere et al., 2010). It can manifest behaviorally in longer response times to process a stimulus (Seymour et al., 2000) and in other ways. A defensive response to a relevant option is also possible if an examinee feels threatened, characterized by increased heart rate and by other signs of arousal. The orienting response to guilty knowledge is hypothesized to be automatic and hard to suppress (Lykken, 1998).

Seymour (2001) proposed a memory-based alternative to Orienting Response Theory called the Parallel Task Set (PTS) model, which explains the “guilty knowledge effect” via response competition. PTS holds that an examinee’s responses to the alternatives of a question of the GKT consist of the following: memory processes, response selection, response preparation, and motor execution. These four components comprise a task set. Two task sets are hypothesized to occur independently and in parallel for each question. The *familiarity task set* occurs quickly and involves automatic priming mechanisms. The *recollection task set*, on the other hand, occurs more slowly, is under conscious control, and draws on cognitive resources. In the case of the relevant alternative, two inconsistent response requests can be received by a particular response processor (e.g., that controlling verbal utterances). In this case, the one received from the familiar task set is for a truthful response while another from the recollection task set is for a deceptive response. One response can also be received while the other response is underway. In both cases, response conflict occurs. Hiding guilty knowledge is postulated to activate conflict resolution, which involves the examinee overriding the familiar response and executing the intended response of denying the guilty knowledge. This model explains the longer response times needed to do so as resulting from the additional processing steps of the recollection task set and the resolution of response conflict. The general insights that the PTS model offers for lie detection are to underscore the centrality of memory processes in deception and truth telling and the fact that the inhibition of a familiar response is often part of deception. Both accounts of the GKT imply that the possession of guilty knowledge manifests in implicit memory measures, which are subtle and hard to hide (Anderson, 2000).

### Four-factor theory

Zuckerman et al. (1981) proposed the influential Four-Factor Theory of deception. It postulates that deception involves (a) generalized arousal, (b) anxiety, guilt, and other emotions accompanying deception, (c) cognitive components, and (d) liars’ attempts to control verbal and non-verbal cues to appear honest. Although these authors speculate that lying imposes greater cognitive load than truth telling, which can result in longer response times, more pupil dilation, and in other signs of load, the theory does not detail the cognitive mechanisms of lying. Still, it highlights the complex, multidimensional nature of deception, and the many types of behavior (e.g., cognitive, physiological, emotional) that are potential cues.

### Interpersonal deception theory

Interpersonal Deception Theory (Buller and Burgoon, 1996; Burgoon and Buller, 2008) focuses on the dynamic, interdependent nature of verbal and non-verbal exchanges between the liar and target (the intended recipient of a deception). Specifically, it describes deception as involving (a) an interaction in which each party of a communicative dyad is monitoring the behavior and responding to cues from the other. (b) The use of strategic deception is postulated to impose a cognitive load on liars absent in truth tellers. Deceivers must consciously manipulate information to create a plausible message, appear honest as they share it, monitor targets’ reactions, and perform other mental tasks. (c) Too many concurrent tasks produce “cognitive overload,” resulting in some behavior going unmonitored. (d) Signs of deception include *uncertainty* and *vagueness* in the detail of a false narrative, *non-immediacy* of responses that involve frequent pausing, and *withdrawal* by sitting away from targets. Liars use *disassociations* to distance themselves from acts of deception, for instance, by describing their actions in a false narrative as going along with the group rather than as resulting from a personal choice.

Four-Factor Theory and Interpersonal Deception Theory posit that a leakage of cues can accompany liars’ strategic control over behavior, especially under high cognitive load. In their review, Zuckerman et al. (1981) found the most reliable leaked cues were the use of self-adaptors (fidgeting hand movements), increased blinking and pupil dilation, heightened voice pitch, and speech errors (grammatical mistakes, slips of the tongue), pausing, and other speech hesitations, and discrepancies between verbal and non-verbal channels. Some cognitive load-inducing techniques we review exploit the fact that under high cognitive load, it is hard for examinees to monitor and control certain channels of behavior, which may maximize the leakage of non-verbal cues as a result.

### Preoccupation model of secrecy

The cognitive load of lies of omission is central to Lane and Wegner’s (1995) Preoccupation Model of Secrecy. It postulates that when individuals keep secrets, for instance, one from a spouse about having been unfaithful, (a) the strategy most often used is *thought suppression*. (“I will stop thinking about having cheated to avoid accidentally blurting it.”) (b) Over time, this ongoing suppression can cause the secret to intrude in the thoughts of the individual. (“I can’t stop thinking about what I did.”) (c) Intrusive thoughts renew attempts at thought suppression. (“I will try harder to block the memory.”) (d) This cycle can escalate to the point that the individual obsesses over the memory long after a secret has been divulged. As does the PTS model, this account notes the difficulty often involved in concealing “guilty knowledge.”

In four studies, Lane and Wegner (1995) found support for steps *a* through *d* and evidence that keeping a secret over time, ironically, increases its accessibility above other memories. Although this model focuses on lies of omission, it has relevance to deception generally. Since most lying involves keeping a secret by withholding some truth, it may help explain the fact that an allocation of cognitive resources is often required to inhibit responding truthfully (Pennebaker and Chew, 1985; Johnson et al., 2004; Kozel et al., 2004; Osman et al., 2009), just as it occurs in thought suppression. Expanding this account, for instance, by integrating it with the PTS model, should increase understanding of when lying requires cognitive resources to inhibit the truth and thereby help pinpoint when cognitive load indices make the most reliable cues to deception. Also, if secret truths become more accessible over time, they may be inadvertently blurted under high cognitive load, an implication of this model for lie detection. Finally, like the PTS model, the Preoccupation model emphasizes memory processes in deception, which in this case is for the active suppression of the truth.

### Self-presentation theory

DePaulo (1992) proposed a Self-Presentation Theory of individuals’ control over their non-verbal behavior to create specific impressions in the minds of others, including deceptive ones. Three cognitive phases are thought to occur. (a) First, an intention to regulate one’s behavior is formed to create a desired impression. (b) Then, the intended self-presentation is translated into non-verbal behaviors. (c) Finally, performance is appraised by the individual, if possible, and lessons are learned for the improvement of future performance. There are obstacles to steps (b) and (c). To note a few, many non-verbal behaviors are hard to monitor, control, or inhibit continuously, such as the expression of basic emotions on the face or the tone of one’s voice (Ekman, 2001). Moreover, the non-verbal behaviors emitted are often more accessible to observers than to those producing them, which makes self-appraisal difficult (DePaulo, 1992).

Although not all self-presentations are deceptive, this account can be regarded as a theory of non-verbal deception. It is consistent with other accounts in that deception involves the intent to misrepresent (Ekman, 2001). Also, like Four-Factor Theory and Interpersonal Deception Theory, it posits that leaked non-verbal cues can signal deception. This account offers more insights than the others on the thought processes involved in looking at a potential lie from the target’s perspective.

### A working memory model of deception

Sporer and Schwandt (2006, 2007) recently offered a Working Memory Model of deception, which is based on Baddeley’s (1992, 2000) influential working memory theory. It too contends that lying imposes a greater load than truth telling due to its heavier processing requirements. Truth telling involves retrieving and reconstructing a memory. When lying, deceivers must invent new stories or modify those available from past experiences or scripts. A deceptive narrative must be plausible and not contradict itself or what the target knows. When no personal memories or scripts are available for lie construction, the working memories of liars will be heavily burdened, reducing capacity for speech production. Liars must also monitor listeners for signs of suspiciousness.

This model’s most unique insights regard the information sources liars use to construct deceptive narratives. It also suggests that cognitive load indices can make reliable cues when examinees are surprised by test items probing details that are likely to be part of the memory of a truthful experience, but not a deceptive narrative.

### The activation-decision-construction model of answering deceptively

The Activation-Decision-Construction Model (ADCM; Walczyk et al., 2003, 2005, 2009) describes answering questions deceptively, which theoretically includes the multiple-choice questions of the GKT. The model analyzes the act into three components. First, a question heard or read activates the truth from long-term memory, usually automatically. Second, based on the activated truth and social context, a decision to lie may be made, usually to advance liars’ interests. Truthful answering will then be actively inhibited, especially for well practiced truths that can proactively interfere with lying. Such response competition is elegantly described by the PTS model. Third, a context-appropriate lie is constructed that must be coherent and plausible. When possible, memories of the truth are altered slightly for the sake of lie plausibility and to minimize the cognitive load of lie construction. Finally, a lie is shared.

Walczyk et al. (2009) expanded the ADCM to account for the rehearsal of deceptive answers. “Deciding to lie” becomes “remember to lie,” with relevant questions and social contexts serving as the memory cues. “Lie construction” becomes “lie recall,” followed by tweaking of the deceptive answers to fit the prevailing social context, both entailing lower loads than spontaneous lying. Responses to questions using the CQT are usually made in less than a second (Lykken, 1998). The expanded ADCM can easily account for this as follows. Either before the exam or during the preview of questions, a deceptive examinee will decide which questions she/he will lie to and will construct deceptive answers. Delivering them during an exam involves cued recall, which typically occurs automatically and quickly (Anderson, 2000).

Several elements of the ADCM have been supported. Walczyk et al. (2003) found, according to self-reports, when participants answered questions deceptively that the truth entered working memory automatically and interfered with lying, consistent with the activation and decision components. Walczyk et al. (2009) demonstrated that individuals lying about well practiced truths had the most difficulty due to a Stroop-like interference. In having participants answer questions about various aspects of their lives either deceptively or truthfully, Walczyk et al. (2005) showed that having to decide to lie adds to cognitive load, and constructing a lie caused greater load than truth telling. One of the ADCM’s implications for lie detection is that when the truth can be pre-activated in examinees and questions are asked that examinees do not anticipate, the processes of deciding to lie and lie construction will manifest as higher cognitive load in liars alone.

### General evaluation of the models/theories

The range of models and theories above illustrates the multifariousness of deception. No single theory could account for all of its cognitive complexity. Generally, these accounts are most relevant to spontaneous (unrehearsed) lying. In such cases, the cues to deception tend to be the richest, including longer response times and more pupil dilation (DePaulo et al., 2003). To be relevant to load-inducing lie detection, they *must be expanded to account for rehearsed deception*, a likely countermeasure. For instance, Interpersonal Deception Theory holds that liars actively monitor their behavior and that of the targets. This may not apply to highly skilled or practiced liars. As suggested by the expanded ADCM, the memory processes of encoding and retrieval will be central to these expansions and become highly automated with practice (Anderson, 2000).

## Lie Detection via Inducing Cognitive Load

Recently an innovative general approach to lie detection has emerged: cognitive load-inducing techniques designed to elicit greater mental effort in liars than in truth tellers (Walczyk et al., 2005; Vrij et al., 2008a). Whereas polygraph-based questioning paradigms rely on elevation in physiological arousal to gauge deception, these use the heightening of indices of cognitive load as the primary cues. Another contrast, although surprising examinees with questions is discouraged in the CQT, given the high rate of false positives that can result (Lykken, 1998), surprising (not shocking) examinees with questions or the task used to access the truth is central to many load-inducing techniques to make it hard to lie. Some techniques below elicit brief responses, as do the CQT and GKT. Others elicit more open-ended responding, such as narratives.

The models and theories above can advance these techniques by showing when and why load indices provide reliable cues (Vrij et al., 2008a). Rather than reviewing all published variations on a common theme, generally only distinctive research-based proposals are discussed, along with their pitfalls and limitations. The results of the experiments testing them show that liars and truth tellers can be classified beyond chance. However, we do NOT discuss the rates of false positives or false negatives for these techniques, because it is far too early in their development to estimate such parameters accurately. This is especially true given that most research is based on college students, not suspects under police interrogation or other authentic samples. Thus, such estimates would be misleading.

### Time restricted integrity-confirmation

Walczyk et al. (2005) proposed a load-inducing technique called *Time Restricted Integrity-Confirmation* (TRI-Con). It is based explicitly on a theoretical account of the differences in mental states between liars and truth tellers, the ADCM. TRI-Con selectively enhances load of liars by surprising examinees with unanticipated questions and by requiring quick responses. These specific guidelines apply to examinations (Walczyk et al., 2005, 2009). (a) Examinees are prompted about the focus of the question set to follow (e.g., “The next 11 questions concern your activities at the time of the crime”). By priming relevant episodic “truths,” prompts reduce examinees’ need to search memory to answer honestly, making cognitive load indices less ambiguous cues that show when a decision to lie and lie construction have occurred. Prompting also reduces the emotional surprise that might be caused by blindsiding examinees with questions probing sensitive issues or incriminating information. (b) Still, the specific questions are not disclosed until asked during an exam, thus surprising examinees cognitively and reducing the rehearsal of lies. (c) Questions are written when possible to be unclear regarding what truths are targeted until they are fully asked. This reduces further examinees’ chance of preparing lies. (d) To obtain clear assessment of the cognitive load needed to answer completely, questions are written to be answerable with one or a few words. (e) Examinees are instructed to answer as quickly as possible to limit further their opportunity to deceive. The high cognitive load of rapid responding to surprise questions may increase cue leakage in the form of voice pitch elevation, pupil dilation, reduced blinking, and long response times because of the limited opportunity for liars to self-monitor and control (Zuckerman et al., 1981; Buller and Burgoon, 1996; Burgoon and Buller, 2008) and may increase accidental blurting of the truth (Lane and Wegner, 1995). (f) Without adequate preparation, liars’ deceptive accounts should be incomplete. Questions are asked and then re-asked along with logically interrelated questions to increase liars’ cognitive load. Contradictions should occur with liars (Granhag and Hartwig, 2008). (g) Behavioral baselines for ground-truth answers are established for all cognitive load indices for comparison with levels of these cues of answers suspected of deception. This practice controls for individual differences in behavioral base rates and improves the accuracy of lie detection (Walters, 1996; Bond and DePaulo, 2006).

Studies have shown the effectiveness of TRI-Con for uncovering deception. Following these guidelines, Walczyk et al. (2005) instructed adults to lie or tell the truth to questions about various aspects of their lives (e.g., employment history, performance on standardized tests). Using response time as the cue, discriminant analyses allowed classification of liars and truth tellers well above chance. Walczyk et al. (2009) tested TRI-Con again by asking participants to lie or tell the truth about their lives and included a rehearsal condition in which participants prepared deceptive answers. The consistency of answers across interrelated questions was added as a cue. Liars and truth tellers were classified up to 89% accurately. The analyses showed that rehearsed deception is detectable. Finally, Walczyk et al. (2012) tested TRI-Con in a forensically relevant context. “Witnesses” observed actual crime videos, then later told the truth or lied rehearsed or unrehearsed about them during interrogation. The cognitive cues were response time, answer consistency, eye movements, and pupil dilation. Discriminant analyses allowed classification of the three conditions 69% accurately, 33% expected by chance.

Despite these promising results, TRI-Con has limitations. For instance, extended narratives given by examinees provide valuable verbal cues to deception (Buller and Burgoon, 1996; Sporer and Schwandt, 2007) that the short answers of TRI-Con are unlikely to tap. Moreover, pupil dilation, blinking rate, voice pitch elevation, and other reliable cues not only measure cognitive load but emotional responses as well (DePaulo et al., 2003). TRI-Con and the techniques to be described may elicit not only cognitive load but also anxiety in examinees. This fact is not problematic when it can be assumed that both anxiety and cognitive load co-vary with deception (Vrij et al., 2010b). Finally, TRI-Con does not allow participants to qualify their answers during the exam, unlike open-ended responses. However, these limitations can be overcome by combining diverse techniques, a possibility discussed later.

### Countermeasures

After new methods of lie detection are introduced, information about them disseminates, and countermeasures are devised. This occurred with the polygraph (Lykken, 1998; National Research Council, 2003) and is occurring with cutting edge approaches, such as functional magnetic resonance imaging (Simpson, 2008; Ganis et al., 2011). Noting this, Walczyk et al. (2005, 2009, 2012) argued that a likely countermeasure against load-inducing lie detection is the rehearsal of a lie, a load reduction strategy (O’Hair et al., 1981; Greene et al., 1985). All research and theory in this area must consider rehearsal. For TRI-Con, other possible countermeasures include examinees intentionally not complying with instructions to answer quickly (e.g., ask that a question be repeated). Likely countermeasures against other load-inducing proposals are discussed as they are presented.

### Asking unanticipated questions and soliciting surprise drawings

Asking questions that examinees do not expect may increase cognitive load. Vrij et al. (2009) instructed pairs of participants to lie or tell the truth about having had lunch together. All pairs then prepared for an interview, which included anticipating likely questions. General and unanticipated questions were later asked, the latter probing minor details like these. What color shirt was worn? Who arrived first? Who sat closest to the door? Inconsistencies in answers to such questions enabled observers to classify liars and truth tellers beyond chance, as did discrepancies in the surprise pictures that the pairs were asked to draw of the layout of the restaurant. Although investigators did not measure the cognitive loads elicited by surprising participants with unexpected questions or the drawing task, we regard both to be load-inducing techniques, because respondents likely had to think a lot when answering or drawing to ensure plausibility and consistently since responding to both was unrehearsed (DePaulo et al., 2003). Recently, Vrij et al. (2012b) observed that truth tellers’ drawings of their workplaces contained more plausible details, especially those involving their coworkers, than liars doing the same.

These results are encouraging. Still, asking unanticipated questions has limitations. Recall that once knowledge of this technique disseminates, liars may include spatial and other obscure details into their deceptive narratives in anticipation of such questions. Second, memory for minor details can easily go unnoticed by truth tellers (Loftus, 2007), making the response “I can’t remember.” plausible when given by liars. The same concerns hold for drawing pictures. Liars can practice drawing them in advance or plausibly deny having noticed spatial details. Still, refinement of these techniques may overcome such concerns.

### Maintaining eye contact with the examiner

Having to maintain eye contact with another can elevate cognitive load and anxiety in liars. In support, Vrij et al. (2010b) directed some participants to lie to interview questions; others told the truth. Some were further instructed to maintain continuous eye contact with the interviewer. Observers of videotapes of the interviews were better at discriminating liars from truth tellers when eye contact was maintained, suggesting that it imposed greater cognitive load and anxiety on liars.

One possible countermeasure is practicing lying while maintaining eye contact with another, which may lessen liar-truth teller differences. Also, sustaining eye contact might prove ineffective with Japanese and those of other non-Western cultures for whom this behavior goes against societal norms. It might induce inordinately high levels of anxiety and be distracting, even for truth tellers (McCarthy et al., 2006). Thus, it is unclear how effective this proposal can be as a general load-inducing technique for distinguishing liars and truth tellers.

### Recounting events in reverse chronological order

The temporal order in which events are recalled can magnify cues to deception. Vrij et al. (2008b) directed half their participants to lie and the other half to tell the truth about what happened during a staged event. Some participants of each condition were further instructed to report events in reverse chronological order. Others reported in chronological order only. More cues to deception emerged and were noticed by observers in the reverse order recounting. The authors noted that recalling in reverse order runs contrary to the typical forward chronological encoding of events and thus imposes a heavy load, especially for liars. Vrij et al. (2012a) extended this technique by asking individuals to lie or tell the truth about a route they took in chronological and in reverse chronological order. More cues to deception again emerged and were noted by observers in the reverse order retelling.

If liars practice lying in reverse chronological order will the cues to deception be as rich? Another likely countermeasure to cover their involvement in crimes, clever perpetrators might base their false alibis on episodic memories of actual events, altering details as needed (Sporer and Schwandt, 2007; Leins et al., 2012). The reverse chronological retelling of these liars might then be similar in cognitive load to that of truth tellers doing the same.

### Dual-tasking (doing two things at once)

Asking examinees to perform a concurrent task during interrogation was a novel approach to load induction tested by Patterson (2010). If lying draws more on attention and working memory than truth telling, then a dual task might interfere more with the former. In this study, truth tellers followed written instructions to go to the university book store, perform specific tasks, and later honestly describe and answer questions about what they did. Liars were shown these instructions but prepared deceptive narratives as if they had been followed, which they later conveyed and answered questions about. During the interview phase, all participants had to perform a concurrent math task. Math response times and accuracies were the dependent measures. Regarding the results, dual task interference was minimal. No liar-truth teller differences were found for math response times, but there was slightly higher math accuracy for truth tellers. Videos of selected interviews were later shown to observers. When interviewees were engaged in a secondary task, observers were slightly more accurate in assessing the veracity of responses and attributed higher loads to liars. This technique is innovative, and more research is needed. However, no theoretical rationale was given for the choice of concurrent task, which may partially explain the weak findings, a theme expanded on later.

### Overall evaluation of load-inducing proposals

Our general impression of the load-inducing approaches to date is that they are innovative and promising. However, it is too soon in their development to accurately gauge their applicability to forensic settings and other real world contexts where detecting deception is vital. Once again, more research is needed on their susceptibility to rehearsal and other countermeasures and on whether the use of such countermeasure is detectable. Also, recall that most of the studies above involved college students who were offered extra credit in exchange for their participation. Their motivation to succeed in their lies was low compared to actual perpetrators trying to persuade detectives with their false alibis or innocent suspects attempting to convince detectives of their innocence. The cognitive loads of guilty liars and innocent truth tellers may both be so high that load-inducing interventions do not differential well between the two (Van Koppen, 2012; Vrij and Granhag, 2012a,b). Research testing these techniques on authentic samples is clearly needed.

## A Theoretical Taxonomy of Cognitive Load-Inducing Lie Detection

Sufficient promising results have been published on load-inducing lie detection (see Vrij et al., 2010a) to justify the proposal of a theoretical taxonomy that can help organize, direct, and advance future validation, refinement, and innovation. It is based on the important distinctions of the type of cognitive load each proposal induces, intrinsic versus extraneous, and how open-ended are the responses each permits in examinees, closed-ended (e.g., short answers, key strokes) versus open-ended (e.g., narratives, drawings).

Two key terms are first defined, both adapted from Cognitive Load Theory (Merrienboer and Sweller, 2005). “Intrinsic cognitive load” refers to the inherent demands on the cognitive resources of attention and working memory needed to lie well. Items 1 through 9 of Table 1 convey some important factors adding to the intrinsic load of lying, organized by whether they relate to preparing a deceptive message or to delivering the message to a target. “Extraneous cognitive load” means any demands on or loss of cognitive resources due to tasks or factors external to the act of lying that makes it more difficult. For example, extreme anxiety in an examinee can decrease available cognitive resources, effectively imposing cognitive load (see Item 10 of Table 1).

**Table 1:** **The cognitive load of lying versus truth telling**.

SOME FACTORS ADDING TO THE COGNITIVE LOAD OF LYING*
**Preparing a deceptive message**
1. Does formation of the lie require that details be kept internally consistent (no contradictory information, Granhag and Hartwig, 2008)?
2. Is the narrative externally consistent (congruent with what the target knows; DePaulo et al., 2003)?
3. Is the narrative detailed enough with multimodal info., a realistic timeline, etc. to convince the target (Vrij et al., 2010a)?
4. Beyond going undetected, are lies based on the deceptive narrative likely to achieve the liar’s goal, for instance, obtaining money from a naïve target (Walczyk et al., 2012)?
**Appearing sincere while delivering a deceptive message to the target**
5. Is the motivation high to lie successfully (Vrij and Mann, 2001)?
6. Not taking credibility for granted, how much monitoring of and control over the self is the liar exercising to appear truthful and to stay in the deceptive role (Zuckerman et al., 1981; Buller and Burgoon, 1996; Vrij et al., 2010a)?
7. How much is the liar monitoring the target’s behavior to, see if the lie is believed (Buller and Burgoon, 1996; Vrij et al., 2008a, 2010a)?
8. Is the truth deeply entrenched, does it elicit strong emotions, or is honest responding well practiced so that proactive interference with deceptive responding occurs (Lane and Wegner, 1995; Morgan et al., 2009; Osman et al., 2009; Walczyk et al., 2009)?
9. Is an adequate deceptive narrative unavailable or is the lie unrehearsed (Vrij et al., 2010a)?
10. Is the liar highly anxious (Eysenck, 1992; Beilock and Carr, 2005)?
SOME FACTORS ADDING TO THE COGNITIVE LOAD OF TRUTH TELLING*
11. Does recalling the truth to working memory require retrieving memories that have not been accessed in a long time or details that have decayed (Anderson, 2000; Wixted, 2004)?
12. Is a lie well rehearsed compared to its corresponding truth (O’Hair et al., 1981; Greene et al., 1985)?
13. Does a truthful response require elaboration or qualification to be accurately understood by the target compared to a corresponding lie (Gombos, 2006)?
14. Does a truthful response require the generation of a novel opinion, judgment, evaluation, attitude, or emotional reaction (DePaulo, 1992; DePaulo et al., 2003; Gombos, 2006)?
15. Is the truth teller highly motivated to be believed (Van Koppen, 2012; Vrij and Granhag, 2012a,b)?

*These lists are not exhaustive.

The extent to which Items 1 through 10 of Table 1 apply to an instance of lying depends on the complexity of its social context (DePaulo et al., 2004). Everyday lies are told without imposing high cognitive loads. Liars typically have little concern about getting caught and rarely monitor their behavior or the targets’ (DePaulo et al., 1996). Thus, few items apply. However, serious lies have greater interpersonal consequences and entail heavier loads (DePaulo et al., 2004; Burgoon and Buller, 2008). More items will apply, especially when lying is spontaneous. On the other hand, skilled or well rehearsed liars telling serious lies may not need to monitor their behavior or the targets’ (Items 6 and 7), instead relying on their fluent delivery to carry them through (DePaulo, 1992).

For cognitive load-inducing lie detection to succeed, it is important to note when truth telling imposes a greater cognitive load than telling a corresponding lie. For instance, Walczyk et al. (2005) found that college students took longer to recall their actual standardized test scores than to lie about them. The bottom of Table 1 lists five factors adding to the cognitive load of honesty. Only when they can be discounted during an examination is lying more likely to manifest in heightened load indices. For instance, questions asked in load-inducing lie detection exams need to be written with Items 11 through 15 in mind so that the cognitive load of lying is higher than for truth telling.

Table 2 provides the full Taxonomy of Load-Inducing Lie Detection and shows where the proposals above and others to be discussed fall within it. Despite the severe limitations of some of them, all proposals are included for the sake of comprehensiveness. A question that should guide their refinement is “*Under what testing conditions are cognitive load indices unambiguous cues to deception?*” To illustrate, such a condition is when “prompting” occurs, which makes cognitive load indices clearer cues by reducing the need for all examinees to search memory for a truth and by reducing the emotional surprise to questions during the exam (Walczyk et al., 2005).

**Table 2:** **A taxonomy of load-inducing lie detection: the type of cognitive load induced and the response open-endedness permitted**.

Intrinsic load	Extraneous load
**Closed-ended responding** (encourages the leakage of hard-to-control non-verbal cues and blurting of the truth)	
TRI-Con	Maintain eye contact
Answering unanticipated short-answer questions about minor details	Dual-tasking-articulartory suppression, n-back task
Implicit personality/attitude tests	Dual-tasking-operate driving simulator or do a concurrent math task
Autobiographical implicit association tests	Have examinee give short answers in front of a mirror
Response time GKT	
**Open-ended responding** (encourages verbal signs of deception, monitoring of self and the target, signs of the attempted control of behavior)	
Have examinee relate surprise narrative	Maintain eye contact
Surprise task of examinees drawing picture of alibi	Have examinees give narrative answers or draw pictures in front of a mirror
Recall events in reverse chronological order	Dual-tasking-operate driving simulator or do a concurrent math task
Recall from a different physical perspective	

### Intrinsic cognitive load-inducing techniques

The proposals under this heading seek to make the act of lying harder by surprising examinees cognitively with test items, with the memory task used to access the truth, or by requiring quick responses. TRI-Con, which elicits closed-ended responses, falls into this category. When examinees have not anticipated the questions, they must decide which ones to lie to and generate deceptive answers on the fly, all adding to cognitive load (Walczyk et al., 2005, 2009). Vrij et al.’s (2008b) proposal of having examinees convey narratives in reverse chronological order fits here, as does instructing them unexpectedly to draw pictures (Vrij et al., 2009). Memories related to the truth are being probed in unusual ways that liars may not have anticipated. Because narratives and drawings can be as elaborate as examinees choose, we consider them to be open-ended. Examinees can pace themselves, monitor, and control their behavior, hopefully causing related cues to emerge (DePaulo et al., 2003).

Although not specifically proposed as a load-inducing technique, Seymour et al. (2000) tested a variant of the GKT with response time as the cue to deception, the *Response Time GKT*, which qualifies as one. Participants partook in a mock crime involving a computer. They also learned two-word phrases related to the crime as well as other two-word phrases later in the experiment. During a subsequent phrase classification task, with instructions to respond *as quickly as possible*, participants were asked to press a key with their right index finger if an item was on the list that had been learned later. All other items required a key press with their left index finger, some of which were from the mock crime. The latter responses were the equivalent of having to conceal guilty knowledge. Responding to guilty knowledge items took about 300 ms. longer than responding to neutral items. Discriminant analyses correctly classified guilty and innocent trials 95% accurately.

Self-report measures of personality and attitudes (e.g., toward members of minority groups) are highly susceptible to deception as some examinees respond to test items to create a false positive image of themselves to obtain jobs and other rewards. Even so, their actual personalities and attitudes are rehearsed through repeated ways of thinking and acting in their daily lives that form strong associations among ideas and emotions in memory (Banse and Greenwald, 2007). Although not proposed as a load-inducing technique either, as an alternative to self-report measures, *Implicit Personality and Attitude Tests* qualify as well. They put examinees under time pressure in responding (Banse and Greenwald, 2007). This increases intrinsic cognitive load by making it harder to deceive. For dishonest examinees, a proactive interference can occur when their true attitudes and personalities conflict with the impressions they want to make, which usually manifests as slower response times for items lied to. Moreover, responses are typically closed-ended, limited to a forced choice between two options. How quickly individuals respond, for example, when instructed to associate the word “good” with the faces of individuals with dark complexions co-presented on a computer screen among many pairings of stimuli, can reveal racism in those responding slowly. This technique has been used successfully in employe selection (Banse and Greenwald, 2007). A variant of it, the *autobiographical Implicit Association Test* (aIAT), was proposed and examined by Sartori et al. (2008). It requires examinees to respond rapidly to test sentences presented one at a time on a computer screen describing autobiographical events that are either true or false for them. Across six experiments, aIAT had accuracies up to 91% in revealing concealed knowledge of true autobiographic events. Still, when examinees use the countermeasure of strategically slowing down when responding truthfully, this accuracy drops dramatically (Verschuere et al., 2009).

We now propose another way of accessing truths that could impose higher intrinsic load on liars and may inspire the development of similar proposals by others. It is based on the encoding-specificity hypothesis (see Anderson, 2000) and part of the “Cognitive Interview,” a well validated set of four memory strategies for assisting individuals in recalling accurately and fully prior events, without inducing memory distortions (Fisher and Geiselman, 1992; Geiselman and Fisher, 1997). First, the interviewer tries to reinstate the physical and mental state of the witnessed event, for instance, by asking the interviewee to form a mental picture of the context of the event and recall how she/he felt. Second, the interviewee is encouraged to recall every detail of an event she/he can, even seemingly insignificant memory fragments. By the third principle, the interviewee is encouraged to recall in a variety of temporal orders. Recall that a variant of this principle was applied to lie detection by Vrij et al. (2008b), specifically recounting in reverse chronological order. Fourth, the interviewee is encouraged to recall from a variety of physical locations, for instance, from how things would have appeared “if you had been looking down on the room where the crime occurred from directly above” or “if you had looked at the room from the perpetrator’s perspective.” The latter principle, too, might be applied to lie detection. If asked to recall from different perspectives, truth tellers should have narratives richer in realistic details that are delivered with fewer hesitations than liars (Sporer and Schwandt, 2007).

### Extraneous cognitive load-inducing techniques

Techniques under this heading seek to induce cognitive load selectively on liars, not by making it harder to lie, but by altering other aspects of the examination procedure or context. “Dual-tasking” was one such proposal considered earlier that is now discussed more deeply. Cognitive scientists have long used this research paradigm to determine when different tasks use a common system or pool of resources (Pashler, 1994; Baddeley, 1996). As a technique for lie detection, it can be used with test items soliciting closed-ended or open-ended responses (Patterson, 2010). Vrij et al. (2008a) suggested that examinees could “recall their stories whilst conducting a computer driving simulation task at the same time” (p. 41). If deception imposes greater load, then the simulation may interfere more with liars, enhancing cognitive cues. To our knowledge, this study has not been done, but is worth testing.

Meyer and Kieras (1997) evaluated various theoretical accounts of multi-task interference. Of them, Unitary Resource Theory is the one that Patterson (2010) and Vrij et al. (2008a) implicitly subscribe to with their proposals. Its basic assumptions are that (a) attentional capacity is a limited general resource that can be assigned to multiple tasks. (b) The amount of attention allocated depends on the demands of the current activities. (c) Under low levels of task load, attention can easily be divided between tasks, not so when either or both of the tasks are difficult. (d) Finally, attention is controllable and can be allocated dynamically. Meyer and Kieras (1997) also review the major criticisms of this account. The one that is most problematic for the proposals above concerns “difficulty insensitivity.” Varying the difficulty of a primary task often does not interfere with a concurrent task, which should occur if both are dependent on a central, limited resource. For instance, difficulty insensitivity was apparently the case with Patterson (2010), who found that lying was minimally disruptive of a concurrent math task.

A powerful framework for understanding multi-task interference effects, which we embrace, is *Adaptive Executive Control* (AEC; Meyer and Kieras, 1997; Meyer et al., 2002). It overcomes the criticisms of Unitary Resource Theory, is instructive regarding what concurrent tasks theoretically should interfere with lying more than truth telling, and is well supported. Five components underlie the framework. (a) It is based on a comprehensive information processing architecture that incorporates all of the known characteristics of human cognition. (b) It is also based on a production system formalism that expresses actions as If-Then rules, which succinctly capture procedural knowledge. The “If” part specifies the conditions under which actions are executed. The “Then” portion specifies the actions in their proper order. (c) Importantly, no assumption of a limited general cognitive resource or capacity is made. (d) Rather, AEC attributes dual task interference to the flexible strategies individuals adopt to fulfill their task priorities as handled by supervisory executive processes. In effect, one task is put on hold while another task higher in priority takes precedence and executes. (e) Finally, AEC explicitly takes account of the constraints in processing imposed by perceptual and motor systems during multi-task performance. For instance, concurrent tasks both requiring verbal responses will naturally interfere. A higher priority utterance will precede the lower priority utterance. Those interested in AEC are referred to Meyer and Kieras (1997) as well as Meyer et al. (2002). To summarize, the major sources of interference are competition between concurrent tasks for the same perceptual or motor response systems or the executive process performing one task before another due to its higher priority given the performer’s goals. If the AEC framework is valid, then the dual-tasking, load-inducing proposals above are unlikely to be effective, because competition for a limited central resource, as they intend, is not the basis of interference.

We now propose a potentially interfering task suggested by the Working Memory Model of deception (Sporer and Schwandt, 2006, 2007). It does not assume a competition for limited attention. Rather, it prevents liars from using a specialized working memory store needed for lying. In research on working memory’s phonological loop, *articulatory suppression* prevents the rehearsal of memory items (Baddeley, 1996), for instance, by instructing participants to continuously repeat a simple word such as “one.” However, repeating a single, familiar syllable might quickly become automated and be minimally disruptive of lying. Having to repeat a sequence of unfamiliar syllables, such as “Bah-Bay-Boo-Bee,” would lessen this problem (Gordon and Meyer, 1987). Will continuously repeating such a sequence interfere more with lying? To test whether, a study can be conducted in which recorded questions are asked through headphones and answers, yes or no, are given non-verbally as keystrokes so as not to impair articulatory suppression. Thus, responding is closed-ended. According to the Working Memory Model, lying requires more access than truth telling to an unencumbered phonological loop for language production. If so, then when articulatory suppression is added, lying should entail longer response times, more pupil dilation, and less blinking than truth telling due to interference caused by competition for this specialized working memory store. Another theoretically based dual task was tested by Ambach et al. (2011) with the GKT: the n-back procedure (deciding whether a stimulus was presented n trials previously). Both tasks were hypothesized to compete for working memory’s central executive. The n-back task enhanced the detection of concealed knowledge as measured by electrodermal activity. Researchers are encouraged to follow these two examples and develop other theoretically based dual task proposals.

Requiring examinees to hold eye contact with the examiner can impose extraneous cognitive load in liars, perhaps by provoking anxiety and an allocation of cognitive resources to monitor the self and the target (Vrij et al., 2010b). As noted previously, this load-inducing technique may not work well with members of some cultures and possibly other segments of the general population for whom maintaining eye contact goes against a social norm (McCarthy et al., 2006). When it is appropriate, it can be used with closed- and open-ended responses. Another way to induce extraneous cognitive load on liars might be to have examinees answer questions while sitting in front of a mirror, which could increase their self-monitoring and the emergence of related cues (Buller and Burgoon, 1996). An examiner would still be needed in the room. This proposal has not been tested.

### Combining intrinsic and extraneous techniques; open- and closed-ended responding

Intrinsic and extraneous load-inducing techniques can be combined in an exam, thereby gaining the advantages of each. For example, examinees can be tested under the intrinsic load-inducing conditions of TRI-Con while following instructions to maintain eye contact with the interrogator (Vrij et al., 2010b). Unanticipated questions about spatial information and other details can be asked. Examinees can also be asked to draw pictures of the physical layout (Vrij et al., 2009).

Closed- and open-ended items can also complement each other (Toris and DePaulo, 1984). Recall that TRI-Con is intended to assess the truthfulness of the short answers given to closed-ended questions, which allows unambiguous assessment of the cognitive load needed to answer using response time, pupil dilation, and other load indices. Moreover, the time pressure on responding may increase leakage of non-verbal cues, which manifest primarily under high cognitive load (Buller and Burgoon, 1996; Burgoon and Buller, 2008) and may increase the chance that a truth is inadvertently blurted (Lane and Wegner, 1995). On the other hand, open-ended questions eliciting narratives can be rich in verbal cues such as vagueness and dissociations (Buller and Burgoon, 1996; Burgoon and Buller, 2008) and in signs of the attempted control of behavior by liars (DePaulo et al., 2003; Sporer and Schwandt, 2006; Vrij et al., 2010a). Interrogators might, for example, have a suspect provide a narrative of an alibis at the time of the crime, followed by a TRI-Con exam with questions probing details of an alibi. If the verbal and control cues from the open-ended portion and the cognitive load indices of the closed-ended questioning all point to deception, strong converging evidence will exist. Also, the more reliable cues to deception used, the more accurate its detection tends to be (DePaulo et al., 2003). It may be worthwhile to combine psycho-physiological and cognitive load cues to enhance lie detection.

## Advancing Cognitive Lie Detection by Avoiding the Pitfalls of the CQT

Four recommendations for researchers and practitioners and their justifications appear below to help the emerging field of cognitive lie detection to avert four of the major weaknesses of the CQT (National Research Council, 2003) and profit from the strengths of the GKT. To recall the criticisms, the CQT is not based on a validated theory and is easily susceptible to countermeasures. Its administration has not been standardized, and the scoring of results is largely subjective.

1. Cognitive load-inducing lie detection techniques should be based on explicitly stated, well-specified, and validated cognitive models of deception (see McCornack, 1997). To many readers this recommendation may be obvious. However, *to date, few load-inducing techniques are based on models or theories that were made explicit in the research reports*. Perhaps the models were implicit in some cases, but this is not helpful to readers wishing to understand the reasons why load-inducing manipulations work or when experimental findings apply to authentic settings. In fact, historically many researchers have sought out reliable cues to deception with minimal regard for their basis in theory (DePaulo et al., 2003). This risks repeating this mistake of the CQT with load-inducing lie detection. Recall that refined cognitive models, supported by data, can illuminate the conditions when load-inducing interventions are likely to succeed, necessary for generalizing the results of experiments to the field (Vrij et al., 2008a). Since deception is multifarious (e.g., verbal, non-verbal, lies of omission) and is driven by many motives (e.g., protect another, conceal wrongdoing, exploit others; DePaulo et al., 2004), no single cognitive account can explain all of its forms (Ekman, 2001; DePaulo et al., 2003).
  The accounts we reviewed can serve as building blocks for narrowly focused models directly applicable to specific authentic contexts like the interrogation room of a police department. To recap those we regard as most applicable, the PTS model specifies the response competition that occurs when examinees falsely deny possessing guilty knowledge. Response competition also likely underlies much of lying, especially when deceiving about well practiced truths. Interpersonal Deception Theory (Buller and Burgoon, 1996; Burgoon and Buller, 2008) highlights the cognitive load of having to monitor the behavior of the self and the target and postulates the leakage of cues under high cognitive load. DePaulo’s (1992) Self-Presentation Theory posits the leakage of cues and delineates between non-verbal behaviors that are easy to control versus those that are not, the latter providing the best cues. Sporer and Schwandt (2006, 2007)’s Working Memory Model elaborates on the sources of information (e.g., scripts, personal memories) used in lie construction. The ADCM is informative about the encoding and retrieval processes related to truth telling, the decision to lie, and lie construction and offers insights regarding the rehearsal of deception (Walczyk et al., 2009, 2012). The Preoccupation Model of Secrecy advances understanding of why cognitive resources are often needed to inhibit truthful responding. Still, these cognitive accounts must be expanded to address the motivation to lie, rehearsal, and other important moderators of cues to deception to be maximally relevant to lie detection (DePaulo et al., 2003).
2. Countermeasures will be devised by deceptive examinees for beating any new method of lie detection as knowledge of it disseminates, nor can this be entirely prevented (Lykken, 1998; National Research Council, 2003; Rosenfeld et al., 2004; Simpson, 2008; Verschuere et al., 2009; Ganis et al., 2011). Researchers and practitioners concerned with cognitive load-inducing lie detection should note that the rehearsal of deception is a serious countermeasure, continue to find ways to minimize it, as well as ways to expose rehearsal when it occurs. Other countermeasures need to be uncovered as well. If this recommendation too seems obvious, it is noteworthy that *few studies testing load-inducing techniques have seriously considered rehearsal or included a rehearsal condition in the research*. We discussed several load-inducing proposals to minimize it such as surprising examinees with test items, with memory tasks, or having examinees respond quickly. Still, rehearsal cannot be prevented completely. Constructing deceptive narratives before an exam as well as anticipating questions and preparing deceptive answers are likely in intelligent, motivated liars (Vrij and Mann, 2001; Vrij et al., 2010a).
  The countermeasure of the rehearsal of deception can be overcome if research can identify “behavioral signatures” that it occurred. More research is needed on the effects of this countermeasure on pupil dilation, voice pitch, response time, blinking rate, and other correlates of cognitive load. Some encouraging findings are that rehearsed liars can have response times falling below those of unrehearsed liars and truth tellers (O’Hair et al., 1981; Greene et al., 1985), as well as reduced eye movements so they can focus on memory retrieval undistracted by the visual environment (Walczyk et al., 2012). Rehearsed liars also have brain activation patterns distinguishable from those of unrehearsed liars and truth tellers (Ganis et al., 2003). The more distinguishing cues that can be developed, the more accurately rehearsed deception can be exposed (DePaulo et al., 2003). Research on the effects of other countermeasures, such as intentionally not complying with instructions to maintain eye context or answer quickly (Verschuere et al., 2009), may reveal distinctive cognitive-behavioral signatures too.
3. Research shows that even law enforcement officers, among other human observers, generally make poor lie detectors (Ekman and O’Sullivan, 1991; Garrido et al., 2004; Bond and DePaulo, 2006, 2008). This is partly because people tend to focus on unreliable cues like gaze aversion or nervousness and miss genuine cues that are often subtle (DePaulo et al., 2003). From police detectives interviewing witnesses to federal agents interrogating suspected terrorists, human lie detectors will be relevant for the foreseeable future. Most of the studies on inducing cognitive load we reviewed have wisely sought to improve the accuracy of human observers, but still report detection rates that are rather low (Vrij et al., 2010a).
  Cognitive load-inducing lie detection involving closed-ended responding offers an alternative to both the CQT and the use of human lie detectors. Recall that administration of the CQT hasn’t been standardized. To elaborate,
  most polygraph testing procedures allow for uncontrolled variation in test administration (e.g., creation of the emotional climate, selecting questions) that can be expected to result in variations in accuracy and limit the level of accuracy that can be consistently achieved (National Research Council, 2003, p. 213).
  The guidelines of TRI-Con and those that might result from refinement of other closed-ended, load-inducing proposals, such as the aIAT and the Response Time GKT, can help standardize lie detection. This does not mean imposing a “predictability” that liars can rely on to foil exams. For instance, unanticipated questions can still be asked. Rather, standardization means following procedures that disambiguate cognitive load indices as cues to deception. TRI-Con, for instance, can be implemented on a laptop computer. Reading and computer skills are not required. Examinees wear a microphone-headset connected to a computer. Questions can be digitally recorded in advance. The assessment of answer response times, pupil dilation, voice pitch elevation, and other cues can be automated with technology now available (Walczyk et al., 2012). Accordingly, we recommend that lie detection exams be developed or further refined to follow standardized cognitive load-inducing procedures and that they be as automated as possible to sidestep the severe limitations of human lie detectors. Human examiners should still be present to oversee administration. Their presence can also induce load selectively on liars as deceptive examinees may feel compelled to monitor them for signs of suspiciousness (Burgoon and Buller, 2008).
4. Appropriate automated analytical procedures should be used to determine the “deceptiveness” or “honesty” of answers. A benefit of standardizing and automating lie detection and assessing the changes in cognitive load between known truthful answers and those suspected of deception is that statistical or other analytic procedures can be used to “decide” if examinees are lying or truth telling. This avoids the subjective scoring of the polygraph (Iacono and Lykken, 1997; Lykken, 1998; National Research Council, 2003). Of course, data must be collected on authentic samples of liars and truth tellers who are highly motivated to convince authorities.

## Conclusion

The polygraph-based CQT lacks a strong scientific basis (Iacono and Lykken, 1997; Lykken, 1998; National Research Council, 2003), unlike the more successful GKT. We believe that cognitive load-inducing techniques are promising alternatives to the CQT, especially if lessons can be learned from the latter. To help them advance, we reviewed many models and theories relevant to the cognition of deception, their implications for lie detection, and evaluated specific proposals for selectively inducing cognitive load on liars, particularly their susceptibility to rehearsal and other countermeasures. The taxonomy proposed classifies these proposals according to the type of cognitive load induced and the breadth of the responses permitted, which may help organize and advance the field by opening up new research areas. Along these lines, new proposals were also suggested. Finally, four recommendations were shared to assist this promising general approach in averting the corresponding pitfalls of the CQT. To date, researchers in this area often have not heeded warning implicit in the report of the National Research Council (2003).

Another contribution of this article is its modest attempt to merge the seemingly disparate fields of “polygraph-based lie detection” on the one hand and “social-cognitive perspectives on deception” on the other. As we have argued, the former has a long history (Lykken, 1998) with many valuable lessons for understanding deception and advancing lie detection. Scholars from each perspective are encouraged to consider research and theory from the other perspective for useful insights and opportunities for cross-fertilization.

Finally, there are several obstacles to societal acceptance of new forensic tools like these cognitive load-inducing lie detection proposals. Will judges, lawyers, solicitors, police officers, victims, suspects, or witnesses accept them? Recalling events in reverse chronological order, drawing pictures, the guidelines of TRI-Con, maintaining eye contact, or answering questions while performing a concurrent task might be rejected for lacking face validity. The most formidable obstacles come with legal tests like the US Supreme Court’s 1993 decision of “Daubert v. Merrill Dow Pharmaceuticals” on the admissibility of scientific evidence in the courtroom. A new method that gives rise to such evidence must (a) have been empirically tested within the applicable field as described in publications in peer-reviewed outlets, (b) have a known potential error rate, (c) have established standards and safeguards for its use, and (d) be widely accepted within the scientific community (Solomon and Hackett, 1996). Much refinement and validation will be necessary to meet these standards. If load-inducing techniques can do so, their acceptance by stake holders will likely follow.

## Conflict of Interest Statement

The authors declare that the research was conducted in the absence of any commercial or financial relationships that could be construed as a potential conflict of interest.
